# Establishment of CTPA Local Diagnostic Reference Levels with Noise Magnitude as a Quality Indicator in a Tertiary Care Hospital

**DOI:** 10.3390/diagnostics10090680

**Published:** 2020-09-09

**Authors:** Hanif Haspi Harun, Muhammad Khalis Abdul Karim, Mohd Amiruddin Abd Rahman, Hairil Rashmizal Abdul Razak, Iza Nurzawani Che Isa, Faeezah Harun

**Affiliations:** 1 Department of Physics, Faculty of Science, Universiti Putra Malaysia, Serdang 43400, Selangor, Malaysia; hanifhaspi@gmail.com (H.H.H.); mohdamir@upm.edu.my (M.A.A.R.); 2 Center for Diagnostic Nuclear Imaging, Faculty of Medicine, Universiti Putra Malaysia, Serdang 43400, Selangor, Malaysia; rashmizal@upm.edu.my; 3 Department of Diagnostic & Applied Health Sciences, Faculty of Health Sciences, Universiti Kebangsaan Malaysia, Kuala Lumpur 56000, Malaysia; 4 Department of Radiology, Hospital Kuala Lumpur, Jalan Pahang, Kuala Lumpur 50586, Malaysia; faeezah.fh@gmail.com

**Keywords:** CT radiation dose, diagnostic reference level, CT pulmonary angiography, noise magnitude, image quality

## Abstract

This study aimed to establish the local diagnostic reference levels (LDRLs) of computed tomography pulmonary angiography (CTPA) examinations based on body size with regard to noise magnitude as a quality indicator. The records of 127 patients (55 males and 72 females) who had undergone CTPAs using a 128-slice CT scanner were retrieved. The dose information, scanning acquisition parameters, and patient demographics were recorded in standardized forms. The body size of patients was categorized into three groups based on their anteroposterior body length: P1 (14–19 cm), P2 (19–24 cm), and P3 (24–31 cm), and the radiation dose exposure was statistically compared. The image noise was determined quantitatively by measuring the standard deviation of the region of interest (ROI) at five different arteries—the ascending and descending aorta, pulmonary trunk, and the left and right main pulmonary arteries. We observed that the LDRL values were significantly different between body sizes (*p* < 0.05), and the median values of the CT dose index volume (CTDI_vol_) for P1, P2, and P3 were 6.13, 8.3, and 21.40 mGy, respectively. It was noted that the noise reference values were 23.78, 24.26, and 23.97 HU for P1, P2, and P3, respectively, which were not significantly different from each other (*p* > 0.05). The CTDI_vol_ of 9 mGy and dose length product (DLP) of 329 mGy∙cm in this study were lower than those reported by other studies conducted elsewhere. This study successfully established the LDRLs of a local healthcare institution with the inclusion of the noise magnitude, which is comparable with other established references.

## 1. Introduction

Computed tomography (CT) has become a necessity in clinical practice to gain beneficial information on diseases and aid the diagnosis of patients. Nevertheless, patients might suffer the side effects of being exposed to a significantly high radiation dose that increases the risk of developing cancer [1]. Several studies have reported that the data acquisition settings, detector configuration, machine quality assurance, patient characteristics, post-processing technique and operator’s skill are factors that expose patients to high doses of radiation in CT examinations [2,3,4,5].

With diverse factors affecting radiation dose exposure, several optimization techniques have been introduced to protect patients [6,7,8,9]. Lately, technological advancements and innovation have helped to significantly reduce radiation dose exposure, which is in line with the as low as reasonably achievable (ALARA) principle. However, the optimization processes must still produce quality images, which are crucial to ensure that clinicians can reach an accurate diagnosis for patients and plan an effective treatment regime. Recently, Christianson et al. introduced an automated technique for estimating the noise level of CT images to facilitate the further improvement of CT protocols [10]. They introduce the term “global noise level”, which characterizes the most frequent noise level in areas of homogeneous tissue. The global noise level, in particular, provides a precise, reliable and automated method for measuring the CT noise for quality assurance programs. Combined with other automated characterizations of imaging performance, the global noise level might offer a promising platform for the standardization and optimization of CT protocols. It is beneficial to ensure the optimization is applied in an appropriate dose without compromising on image quality.

Automatic tube current modulation (ATCM) is one of the most promising tools to compensate for radiation exposure in the specific patient’s attenuation factor [11]. By modulating the tube current along the Z-axis of the patients, it allows a CT dose optimization specifically on the thoracic and abdominal areas. The newly developed optimized technique, including current modulation along the X and Y-axes, and tube potential adaptation with dual-energy imaging protocol or monoenergetic algorithm, has been acknowledge for reducing the CT dose significantly [11,12]. Meanwhile, the noise index (NI) is set manually based on the patient’s body size as adapted by in the United States-manufactured General Electric CT scanner [13]. In another way, the ATCM might produce a consistent image quality of a patient either by increasing the radiation exposure for a large-sized patient or limiting the exposure for a small patient. Thus, the radiologists’ concern about producing acceptable images for diagnoses by the ATCM system has been largely solved [14,15,16].

In 1990, the International Commission on Radiological Protection (ICRP) emphasized the importance of determining the diagnostic reference level (DRL) towards further investigation for optimization [17,18,19]. The DRL also serves as a standard for monitoring doses as it can indicate if the exposure is considered too high compared with various healthcare institutions in a region. The standard quantities to publish the DRL in CT examinations are the CT dose index volume (CTDI_vol_) and dose length product (DLP). However, through the years, a misconception of the DRL has been widely applied in clinical institutions. The DRL has been mistakenly considered as the threshold dose, regardless of patient size and clinical indications [17,20,21]. As a result, some CT examinations, especially on large patients, have resulted in compromised images due to poor optimizations, and repeated scans had to be performed, thereby increasing unnecessary radiation exposure. The DRL should only be considered as an indicator for investigation levels of optimization, and it represents good clinical practice for groups of patients, but not for an individual patient [21].

The US National Council on Radiation Protection and Measurement (NCRP) has recommended that the achievable dose values should be set at a median value instead of the mean, as practiced [22,23]. The DRL usually established at the 75th percentile of the median values attained from various representative centers. It should be noted that the DRL values obtained are highly dependent on the state of practice implemented at a specific examination or institution. Previously, the radiation dose in CT pulmonary angiography (CTPA) examinations could be reduced when applying a test bolus method, rather than implementing bolus tracking, thus causing significant changes in the DRL values [24]. It is appropriate to standardize the same procedure to be introduced at a certain point of the DRL. The DRL established using low-end CT scanners may not be appropriate for institutions that carry out CT procedures with the latest high-tech scanners. A low DRL may be enough to produce a sufficient image quality comparable to a high technology scanner.

As in previous work expanding the DRL concept with regard to image quality, this study also targets the same objectives but with a different approach using CTPA examinations as a model [20]. Since CTPA is a widely used first line technique for diagnosing patients with suspected pulmonary embolism (PE), thus the reduction in radiation dose associated with CTPA is of paramount importance from the clinical perspective to access a dose survey regarding this examination [4,25]. Generally, smaller patients only require a small amount of radiation exposure to attain a good image quality compared with larger patients. The rationale to integrate the size dependent DRL with regard to the image quality level is essential to ensure a better optimization strategy across a wide range of patient sizes. Hence, the purpose of the study was to evaluate the median values, ranges, and reference range for both the dose metrics and image quality (noise magnitude) based on patient sizes to arrive at meaningful DRLs. The results are also compared with CTPA practices in other countries. As encouraged by the International Atomic Energy Agency (IAEA), this study is an adequate first step to deliver a comprehensive process of optimization, specifically in CTPA examinations.

## 2. Materials and Methods

### 2.1. Patients

This retrospective study was approved by the Medical Research and Ethics Committee (MREC) of the Ministry of Health Malaysia (MOH), which waived the need for patient consent (approval ID: NMRR-18-3088-44138; date: 13 March 2019). The patients comprised 127 adults (55 males and 72 females) who underwent CTPA at a tertiary hospital in Kuala Lumpur, Malaysia. This study was based on the recommendations of ICRP Publication 135, where at least 30 subjects were required for establishing a DRL in a specific patient group [17]. The data were gathered between January 2019 and May 2019, and all patients were at least 18 years old. All examinations were performed using a Philips Brilliance iCT 128-slice CT scanner (Koninklijke Philips NV, Amsterdam, the Netherlands), with the images reconstructed using the DICOM software, OsiriX version 3.8 (Pixmeo SARL, Switzerland). All subjects had been diagnosed with PE based on their history and clinical symptoms.

### 2.2. CT Parameters

CTPA was performed according to the healthcare institution’s protocol. The bolus tracking (B-T) technique was used to enhance the optimal intraluminal contrast, with a trigger level between 70 and 110 HU and a delay time between 10 and 14 s based on patient habitus. In each patient, the contrast media was conducted, with a flow rate of 5 mL/s followed by a saline chaser (50 mL; flow rate 5 mL/s). All radiographers were well trained in performing the procedure as they all had more than 3 years’ experience.

**Figure 1:**
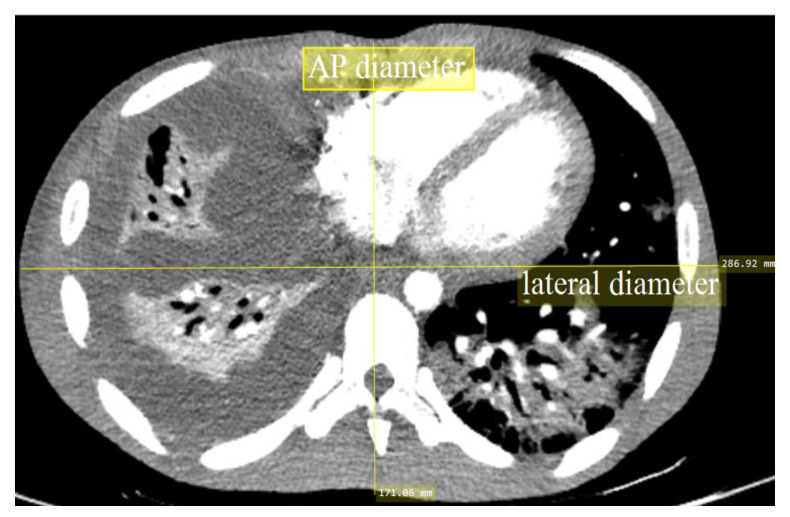
A patient’s anteroposterior (AP) measurement at the mid-slice of the 3D computed tomography (CT) images.

All relevant data, such as the tube potential (kV), tube current (mA), rotation time, pitch factor, CTDI_vol_, DLP, gender, and anteroposterior (AP) body length, were documented from the CT system console in designated survey booklets. Each subject’s body habitus was represented by the AP body length at the middle slice of the scanning area (Figure 1). The AP body length was selected because the ATCM system of the scanner could only modulate the *Z*-axis alongside the patients. The lengths in this study cohort were then divided into three groups: P1 (14 to 19 cm), P2 (19 to 24 cm), and P3 (24 to 31 cm). Only examinations using a PE protocol were included in this study, and multiphase examinations were excluded.

### 2.3. Risk Assessments

The CTDI_vol_ and DLP values were evaluated using CT-EXPO software Version 2.3.1 (SASCRAD, Bucholz, der Norheide, Germany) based on the data recorded in the survey booklet. This provided a promising software-rendered comprehensive evaluation of the radiation doses of various scanner models, manufacturers, and parameters. The software used the Monte Carlo simulation and mathematical phantoms to calculate the effective dose (E) for each patient from the DLP values. The effective dose represented the risk level of a patient developing cancer after being exposed to radiation in a CT procedure. Basically, the individual E was calculated based on the following equation:

(1) E=DLP×k

where k is the values of 0.020 mSv∙mGy^−1^∙cm^−1^ used in the CT-EXPO. The weighting factor was referring from the ICRP 103 (2007).

### 2.4. Image Quality Evaluation

**Figure 2:**
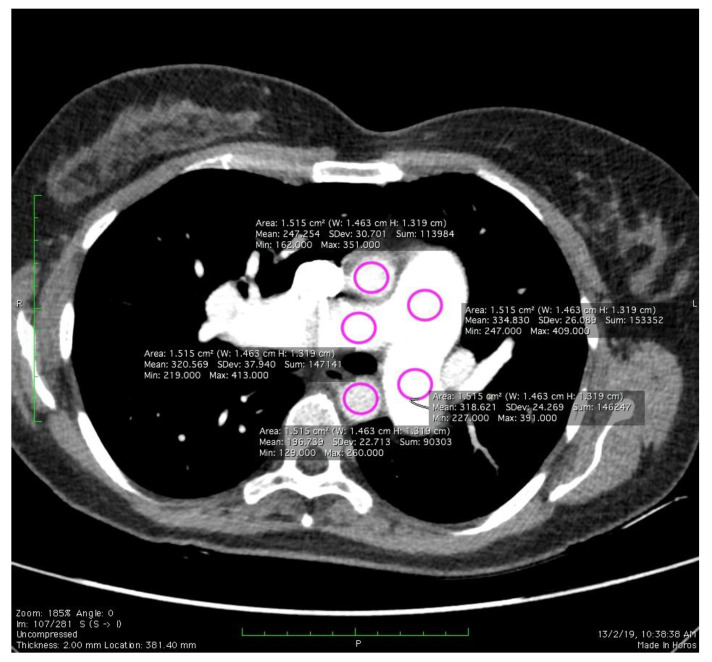
Placement of the regions of interest (ROI) for the noise magnitude calculation.

The noise values of the CTPA images were determined by measuring the magnitude (SD) at the pulmonary artery. The assessment was done by placing the circular regions of interest (ROI) along the main pulmonary artery (MPA), right pulmonary artery (RPA), left pulmonary artery (LPA), ascending aorta (AA), and descending aorta (DA) (Figure 2). Noise may arise from a variety of sources, such as photon or thermal fluctuations, and may even rely on the signal. Noise in CT is normally presented as a standard deviation, σ and formulated as the equation below:

(2) $$\mathrm{Noise}\left(\sigma \right)=\sqrt{\frac{\sum {\left(x_{i}-\bar{x}\right)}^{2}}{n-1}}$$

where *x* is a CT number in the Hounsfield unit (HU), $\bar{x}$ is the average CT number inside the ROI, and n is a total HU inside the ROI. If PE was present, ROI circles were carefully drawn without incorporating the embolic material.

### 2.5. Statistical Analysis

The descriptive statistics were reported in this study as fractions and means with standard deviations. All data were entered SPSS V17.0 (SPSS, version 17.0 for Windows, Chicago, IL, USA) for statistical analysis. The Shapiro–Wilk test was used to determine the normality of the data. Applying the Shapiro–Wilk test to a total of 127 patients (95% confidence level), we found that the measured values of all parameters were not normally distributed. The Kruskal–Wallis test was used, and *p*-values < 0.05 were considered statistically significant.

## 3. Results

**Figure 3:**
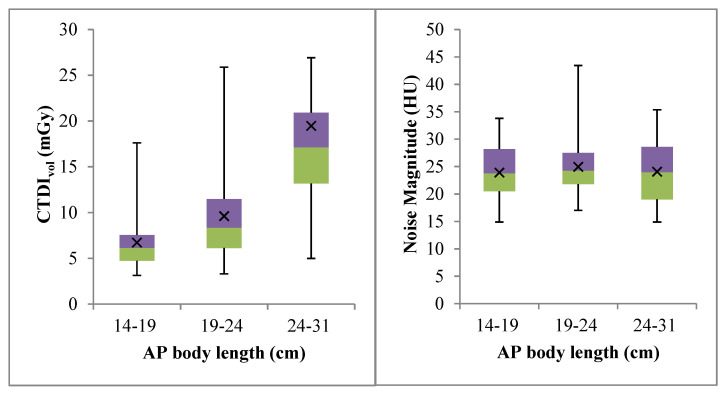
Comparison between the dose exposure and image quality performance in different patient AP body length groups.

**Figure 4:**
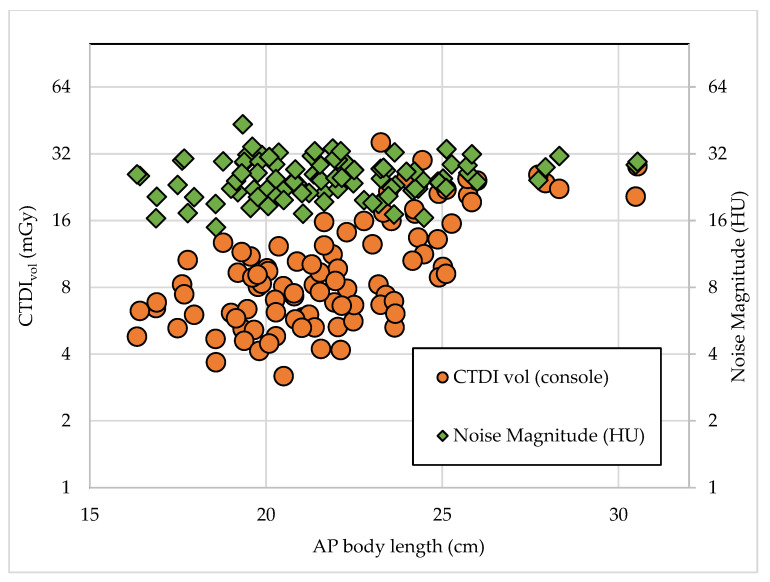
Relationship between the CT dose index volume (CTDI_vol_) and noise magnitude with the AP body lengths of the subjects.

Table 1 provides the baseline patient characteristics according to gender in CTPA examinations using tube potentials of 100 kVp and 120 kVp. However, other protocols were similar, as stated in Table 2. The mean CTPA radiation dose measurements are listed in Table 3. The highest radiation dose for the CTDI_vol_, DLP, and E were in the 120 kVp group, and such a voltage tended to be used on patients in larger AP body length groups (P2 and P3). On the contrary, the 100 kVp group showed the lowest exposure values with most of the patients belonging to the smallest AP body length group (P1). Table 4 provides descriptive statistics for the noise magnitude in this study. The noise magnitude was not statistically different between the different tube potential settings and AP body lengths. Overall, the noise magnitude surrounding the pulmonary arteries was in the range of 14.90 to 43.44 HU. Table 5 and Figure 3 and Figure 4 show the relationships between the CTDI_vol_ and noise values for different AP body lengths. The medians of the CTDI_vol_ and DLP values in this study were compared with other studies in Table 6. The CTDI_vol_ and DLP were lower than has been reported in previous studies.

## 4. Discussion

This study proposed a novel way to establish the LDRL of the CTPA examination with respect to noise magnitude as a quality standard. The application of ATCM in CTPA led to a significant increase in all the dose descriptors in Table 3, as the patients’ AP body lengths increased. A higher tube potential would generate an X-ray beam with a greater frequency, thus increasing the radiation dose [31].

As recommended in ICRP 135, the necessity of including the noise reference levels to establish the local DRL is presented in this study. A recent study by Ria et al., 2019 reported the first approach of a new concept for DRLs in an abdominal CT examination [20]. However, our findings showed a negligible variation in the noise magnitude, which was not reported in their research. While the tube current was modulated by the ATCM system, a broadly constant trend of noise magnitude was observed even though the radiation exposure increased for larger-sized patients. Hence, the institution’s current CTPA protocols could produce a consistent image quality in patients of various sizes.

The justification and optimization of CT according to the principle of radiation protection is a cornerstone for diagnostic procedures. Scanner manufactures, radiographers, medical physicists, and radiologists have roles to ensure that CT examinations are safe to perform. Technological advancements have improved the DRLs of CT scanners and reduced the noise magnitude, as supported by a recent finding [21]. Hence, the establishment of scanner dependent DRLs is pertinent where technological advancements have resulted in significant variation in patient doses. A recent development known as the iterative reconstruction algorithm could provide superior image quality with a lower radiation dose exposure compared with the older models of CT scanners [4,32,33]. Thus, the DRL should be constantly reviewed whenever healthcare institutions upgrade their CT scanner models to the latest in the market. All DRLs should be specifically used for the particular generation of the scanners that they were established on.

There are several contributors that account for our study having the lowest CTDI_vol_ and DLP values among similar published studies. Our finding only represents a single institution and data from one scanner model, which is considered to be a high-end scanner (128 slices). Meanwhile, other studies involved multiple institution and scanner models. Multiple scanner models with different acquisition protocols and technology advancements produced a different radiation output, as well as varying DRLs. In this study, the median CTDI_vol_ and DLP values were compared with the DRL data from studies in other countries [26,27,28,29,30]. The DLP value had been slightly lower than the other established reference. This difference could be due to the axial or helical acquisition scanning selection mode, the CT scanning parameter settings or the different CT scanner manufacturers used. This argument shows the need to provide the scanner dependent DRLs instead of combining all the dose report data, regardless of the type of scanner, to develop DRLs as a common practice.

Theoretically, scanning could be controlled by increasing or decreasing the pitch factor, where the CTDI_vol_ received by patients would be reduced as the pitch factor increased, but at the expense of image quality [34,35]. However, for high-end MDCT scanners, such as second or third generation dual-source CT, a high temporal resolution ensures a reasonable diagnostic performance even with a high pitch factor [36,37,38]. Modern scanners were developed to cover a wider beam collimation range per rotation of the CT X-ray tube. These would reduce the scanning time and radiation dose received by the patient without compromising the image quality. The DLP was highly dependent on the scan length and range of the patient, where the increase in both factors would also increase its value.

There were some limitations to this study. This study is limited by only one type of examination. Further work should seek to expand to other CT examinations to establish a comprehensive set of DRLs. Moreover, this study determined the DRL using only a scanner from a single manufacturer, which did not reflect the performance of other models by other manufactures and their technological advancements. Only a single study center is measured, so this study does not portray a dose exposure trend in other institutions that use dissimilar CTPA practices, CT technology and scanning parameters. The DRL established herein was based on median values while most of the established DRLs were based on the third quartile (75th) values, which may have been biased. Lastly, the necessity for the CT protocol study had been suggested for our institution to compensate for the diagnostic performance of cross-sectional images with exposure parameters. Despite these limitations, we present our preliminary findings, which can be useful for optimizations in local institutions, while waiting for an updated study on the establishment of comprehensive DRLs by local authorities.

## 5. Conclusions

In conclusion, the dose metric with a higher tube potential and patient size was found to increase. The image quality was nevertheless unchanged even though the size of the patient increased. The observed DRL levels here are lower than those reported in other countries. Our institution has been concerned with providing detailed DRL guidance with a continuous optimization strategy, particularly in CTPA and expanding DRL work with various available CT scanners.

## Figures and Tables

**Table 1:** Data on the baseline characteristics based on gender.

Baseline Characteristic	100 kV Group (*n* = 74)		120 kV Group (*n* = 53)	
	Male	Female	Male	Female
No. of examination (*n*)	34	40	21	32
AP body length (cm) *	19.79 ± 2.61	20.81 ± 2.52	23.38 ± 3.58	23.36 ± 2.47
Age *	49.36 ± 17.84	48.00 ± 20.29	48.19 ± 15.97	53.58 ± 18.12

* (mean ± SD).

**Table 2:** Data on the scanning acquisition in computed tomography pulmonary angiography (CTPA) examinations.

Scanning Parameter	Values
Tube Voltage (kV)	100 and 120 kV
Tube Current (mAs) *	186.64 ± 81.84
Scan Range (mm) *	277.44 ± 88.03
Pitch Factor	0.798
Beam Collimation (mm)	0.625 × 64
Slice Thickness (mm)	1
Reconstruction Interval (mm)	0.5
Rotation Time (s)	0.50

* (mean ± SD).

**Table 3:** Data on the scanning acquisition in CTPA examinations.

Tube Potential (kV)/Group	100			120		
	P1	P2	P3	P1	P2	P3
No. of examinations (n)	29	38	7	3	26	24
CTDI_vol_ (mGy) *	5.63 ± 2.11	7.20 ± 2.17	11.60 ± 5.71	9.27 ± 3.35	15.44 ± 6.81	20.44 ± 5.43
DLP (mGy.cm) *	227.71 ± 85.79	270.17 ± 91.48	465.20 ± 261.09	373.09 ± 104.37	577.71 ± 219.51	799.84 ± 255.25
Effective Dose (mSv) *	4.34 ± 1.95	5.43 ± 2.01	8.88 ± 3.68	7.47 ± 2.50	11.75 ± 5.04	15.47± 4.84

* (mean ± SD); P1 = 14 to 19 cm; P2 = 19 to 24 cm; P3 = 24 to 31 cm.

**Table 4:** Noise magnitude measurements according to the AP body length groups.

Tube Potential (kVp)/Group		100			120		
		P1	P2	P3	P1	P2	P3
No. of examinations (*n*)		29	38	7	3	26	24
Noise Magnitude (HU)	Range	14.90–33.80	17.03–43.44	18.28–33.25	21.46–29.81	17.17–34.46	14.90–35.36
	Median	24.10	24.67	23.97	22.27	22.16	24.11
	Mean	23.85	25.91	24.46	24.51	23.58	23.96

P1 = 14 to 19 cm; P2 = 19 to 24 cm; P3 = 24 to 31 cm.

**Table 5:** Local diagnostic reference levels (DRLs) and noise reference levels.

AP Body Length (cm) *	14–19	19–24	24–31	*p*-Value
Dose Reference Level (mGy)	6.13	8.35	21.40	<0.001
Dose Reference Range (mGy)	4.73–7.55	6.13–11.50	13.70–25.20	n.a.
Noise Reference Level (HU)	23.78	24.26	23.97	0.703
Noise Reference Range (HU)	20.49–28.18	21.80–27.50	19.02–28.60	n.a.

* median value.

**Table 6:** Comparison of DRLs in CTPA with other studies.

Established MSCT DRLs	Dose Descriptor	
	CTDI_vol_ (mGy)	DLP (mGy∙cm)
This study	9	329
Saudi Arabia (2014) [26]	18	480
Netherlands (2012) [27]	10	350
Ireland (2012) [28]	13	432
United Kingdom (2011) [29]	13	441
Switzerland (2010) [30]	15	467
